# Earlywood Vessels in Black Ash (*Fraxinus nigra* Marsh.) Trees Show Contrasting Sensitivity to Hydroclimate Variables According to Flood Exposure

**DOI:** 10.3389/fpls.2021.754596

**Published:** 2021-10-14

**Authors:** Jacques Clément Tardif, Susanne Kames, Alexandre Florent Nolin, Yves Bergeron

**Affiliations:** ^1^Centre for Forest Interdisciplinary Research (C-FIR), Department of Biology/Environmental Studies and Sciences, University of Winnipeg, Winnipeg, MB, Canada; ^2^Institut de Recherche sur les Forêts, Université du Québec en Abitibi-Témiscamingue (UQAT), Rouyn-Noranda, QC, Canada; ^3^Centre d'Étude de la Forêt, Université du Québec à Montréal (UQAM), Montréal, QC, Canada; ^4^Department of Botany, University of Manitoba, Winnipeg, MB, Canada

**Keywords:** ring-porous wood, earlywood vessels, spring floods, flooded and non-flooded habitats, eastern boreal Canada, dendrohydrology, dendroclimatology

## Abstract

In recent years, the utility of earlywood vessels anatomical characteristics in identifying and reconstructing hydrological conditions has been fully recognized. In riparian ring-porous species, flood rings have been used to identify discrete flood events, and chronologies developed from cross-sectional lumen areas of earlywood vessels have been used to successfully reconstruct seasonal discharge. In contrast, the utility of the earlywood vessel chronologies in non-riparian habitats has been less compelling. No studies have contrasted within species their earlywood vessel anatomical characteristics, specifically from trees that are inversely exposed to flooding. In this study, earlywood vessel and ring-width chronologies were compared between flooded and non-flooded control *Fraxinus nigra* trees. The association between chronologies and hydroclimate variables was also assessed. *Fraxinus nigra* trees from both settings shared similar mean tree-ring width but floodplain trees did produce, on average, thicker earlywood. Vessel chronologies from the floodplain trees generally recorded higher mean sensitivity (standard deviation) and lower autocorrelation than corresponding control chronologies indicating higher year-to-year variations. Principal components analysis (PCA) revealed that control and floodplain chronologies shared little variance indicating habitat-specific signals. At the habitat level, the PCA indicated that vessel characteristics were strongly associated with tree-ring width descriptors in control trees whereas, in floodplain trees, they were decoupled from the width. The most striking difference found between flood exposures related to the chronologies' associations with hydroclimatic variables. Floodplain vessel chronologies were strongly associated with climate variables modulating spring-flood conditions as well as with spring discharge whereas control ones showed weaker and few consistent correlations. Our results illustrated how spring flood conditions modulate earlywood vessel plasticity. In floodplain *F. nigra* trees, the use of earlywood vessel characteristics could potentially be extended to assess and/or mitigate anthropogenic modifications of hydrological regimes. In absence of major recurring environmental stressors like spring flooding, our results support the idea that the production of continuous earlywood vessel chronologies may be of limited utility in dendroclimatology.

## Introduction

In recent decades, developments in tree-ring research have facilitated the quantification of the anatomical traits of tree-ring and contributed to the promotion of wood anatomical research. The recent assembly of microtomes adapted for tree-ring research (Gärtner and Nievergelt, 2010; Gärtner et al., 2014, 2015a) and the concomitant publications on the art of making wood thin sections (Gärtner and Schweingruber, 2013; Gärtner et al., 2015b; Tardif and Conciatori, 2015; von Arx et al., 2015, 2016) added to this trend. High-definition digital cameras and scanners have made it easier and faster to acquire images of tree rings at both macroscopic and microscopic scales. Image analysis procedures and software, e.g., Canny edge algorithm—Canny, 1986, ImageJ2—Rueden et al., 2017, WinCell—Régent Instruments Inc, 2005, ROXAS—von Arx et al., 2013 and von Arx and Carrer, 2014, and CATS—Land et al., 2017, have all eased measuring and analyzing wood cell dimensions. Methodological reviews and recipe-type publications have also contributed to systematize many procedures (García-González and Fonti, 2006, 2008; Fonti et al., 2009a, 2010; Scholz et al., 2013; Gärtner et al., 2015b; García-González et al., 2016; von Arx et al., 2016).

Tree rings and their intra-annual anatomical variations form important environmental archives. Older and recent reviews have emphasized the utility of many anatomical features in both coniferous and broadleaf species as they relate, among others, to extreme climate events (Wimmer, 2002; Ballesteros-Cánovas et al., 2015; Bräuning et al., 2016; De Micco et al., 2016; Tardif et al., 2020, 2021). Many tree-ring features like frost rings, false rings [or intra-annual density fluctuation (IADF) in Europe], and flood rings are easily identified macroscopically. However, as the visual cues used to identify these anomalies often weaken alongside the intensity of the stressor, e.g., climate signal, microscopic identification with detailed anatomical analyses may often remain the only detection pathway. For example, Waito et al. (2013) reported that many weakly developed frost rings could not be observed macroscopically but only microscopically after thin sections preparation. The current discussion surrounding light rings (macroscopic) and blue rings (microscopic) [see Crivellaro et al. (2018) and Tardif et al. (2020)] also echoes the recent developments and applications of detailed wood anatomical research in dendrochronology.

### Earlywood Vessels and Riparian Ring-Porous Tree Species

In dendrohydrology, the utility of vessel anatomical traits in riparian ring-porous species has long been recognized (Ballesteros-Cánovas et al., 2015; Tardif et al., 2021). In response to spring flood events, many ring-porous tree species produce well-developed flood rings in the submerged portion of their stem, i.e., when inundation coincides with active earlywood vessel formation (St. George et al., 2002; Copini et al., 2016). Flood rings are not to be confounded with the anatomical response of ring-porous species to mechanical injuries associated with flooding (Tardif et al., 2021). Flood rings can easily be distinguished macroscopically (visually) due to their distinct vessel characteristics compared with typical tree rings. Novice dendrochronologists after receiving proper training can reliably identify them (Tardif et al., 2021). Flood rings were characterized through earlywood vessels with the abnormally small lumen in both white and green ash (*Fraxinus americana* L. and *Fraxinus pennsylvanica* Marsh; Yanosky, 1983), in pedunculate oak (*Quercus robur* L.; Astrade and Bégin, 1997; Sass-Klaassen, 2009; Sass-Klaassen et al., 2010; Copini et al., 2016), in bur oak (*Quercus macrocarpa* Michx, St. George and Nielsen, 2000, 2002, 2003; Wertz et al., 2013; Therrell and Bialecki, 2015), in overcup oak (*Quercus lyrata* Walt, Therrell and Bialecki, 2015; Meko and Therrell, 2020), and black ash (*Fraxinus nigra* Marsh., Tardif et al., 2010, 2021; Kames et al., 2016; Nolin et al., 2021a,b, accepted).

The majority of the aforementioned studies used macroscopically identified flood rings and their annual frequency as pointer years to detect exceptional flood events. In some studies (Astrade and Bégin, 1997; St. George and Nielsen, 2002; St. George et al., 2002), short time series derived from earlywood vessel cross-sectional lumen area were made to quantitatively define these flood-signature rings. The study of Meko and Therrell (2020), in addition to visual identification of flood rings, measured the width of the first row of earlywood vessels in *Q. lyrata*. Both proxies were strongly related to spring river flooding. In contrast, the study of Astrade and Bégin (1997) observed in *Q. robur* that vessels in the first row were of normal size during the flood of 1983 but that they were followed by several rows of abnormally narrow vessels. In these two studies, the location of abnormal vessels (first row or subsequent rows) may be attributable to different flood dynamics (timing and duration) and these differences remind us of the need to make thoughtful choices when developing research protocols. Some studies using floodplain ring-porous species did not report the presence of flood rings nor decisive associations between earlywood vessel dimensions and current year floods (Gričar et al., 2013; Tumajer and Treml, 2016; Koprowski et al., 2018). Results from these studies may relate to specific flood regimes but also to sampling at 1.3 m above ground level and/or neglecting small vessel measurements. At last, recent work has focused on developing long annually resolved measurement series of earlywood vessel attributes (Tardif et al., 2010; Kames et al., 2016) leading to a multi-century spring discharge reconstruction for northeastern boreal Canada (Nolin et al., 2021a).

### Earlywood Vessels and Non-riparian Ring-Porous Tree Species

In non-riparian ring-porous tree species, no earlywood vessel abnormalities have to our knowledge been utilized to the extent of flood rings in riparian species. No comparable features to flood rings have been macroscopically used to identify extreme events despite studies indicating that abnormally small cross-sectional earlywood vessel lumen areas may result from insect defoliation (Huber, 1993; Asshoff et al., 1999; Thomas et al., 2006), severe drought (García-González and Eckstein, 2003), and forest fires (Kames et al., 2011). The study of Fletcher (1975) mentioned using abnormally small earlywood vessels to cross-date oak panels. Nonetheless, the hydroclimatic signal in ring-porous species growing in mesic and xeric habitats have often been said to belong essentially in the largest earlywood vessels (García-González and Fonti, 2006; Fonti et al., 2009a; García-González et al., 2016), while small earlywood vessels constitute noise (García-González and Fonti, 2006; González-González et al., 2015). This is largely contrasting with riparian species in which the hydroclimatic signal resides particularly in small earlywood vessels (St. George et al., 2002; Kames et al., 2016; Nolin et al., 2021a). Studies [for example, refer to García-González and Fonti (2006), Fonti et al. (2009a), and González-González et al. (2014)] recommending that small earlywood vessels, e.g., <6,000 or <10,000 μm^2^, be omitted to speed-up the development of ring-porous earlywood vessel chronologies “without any signal lost” have had an undetermined impact on knowledge acquisition in the field and especially when the environmental signal lies in small vessels.

Nonetheless, earlywood vessel cross-sectional lumen area in non-riparian trees have been shown to be affected by temperature and water availability during the previous growing season, the dormant period and/or the current growing season, wherein the prevalence of any factor being dependent on the requirements of species, climate regions, and microsite conditions (for examples, see Villar-Salvador et al., 1997; García-González and Eckstein, 2003; Corcuera et al., 2004, 2006; Fonti and García-González, 2004, 2008; Tardif and Conciatori, 2006; Fonti et al., 2009b; Alla and Camarero, 2012; González-González et al., 2015; Pritzkow et al., 2016; García-González and Souto-Herrero, 2017; Pérez-de-Lis et al., 2018; Zhu et al., 2020). The study of García-González et al. (2016) provided an informed review on the topic. Many studies that analyzed vessel attributes and ring width showed that earlywood vessel cross-sectional lumen area contained unique climate information but that this variable was not very sensitive (Fonti and García-González, 2004, 2008; Tardif and Conciatori, 2006; Alla and Camarero, 2012; García-González et al., 2016; Pritzkow et al., 2016), or was deemed to be too weak compared with tree-ring width features to be used in dendroclimatological reconstruction (Tardif and Conciatori, 2006; Alla and Camarero, 2012). Current climate reconstructions using vessel traits from *Q. robur* report fairly low *r*-square values ranging from 0.22 (period 1961–2011; Davis and Loader, 2020) to 0.31 (period 1951–2010; Pritzkow et al., 2016). These values largely contrast with those reported for floodplain environments. The study of Nolin et al. (2021a) explained more than 69% of the variance in instrumental discharge using *F. nigra* earlywood vessel chronologies over the period 1916–2016.

### Objectives and Hypotheses

The general objective of this study was to provide a thorough comparison of earlywood vessel major anatomical traits in *F. nigra* trees growing under the same general climate but with contrasting exposure to spring floods. Tree-ring widths and earlywood vessel chronologies were developed from wood samples collected from *F. nigra* trees growing in flooded and non-flooded sites. In addition to the traditional measurements of earlywood, latewood, and total ring width, the number of vessels and their cross-sectional lumen area above 800 μm^2^ was measured. It was hypothesized that chronologies developed from the non-flooded site are independent of those developed from the flooded site, thus, confirming the uniqueness of the flood signal to floodplain trees. Given that water availability differs in the two study areas and throughout the growing season, it was further hypothesized that earlywood vessels in each situation will provide different hydroclimatic signals due to their distinct exposure to water and drought stresses. Finally, it was hypothesized that earlywood vessel chronologies developed from non-flooded trees would bear little potential for hydroclimate reconstruction compared with their floodplain equivalents.

## Materials and Methods

### Study Area

**Figure 1:**
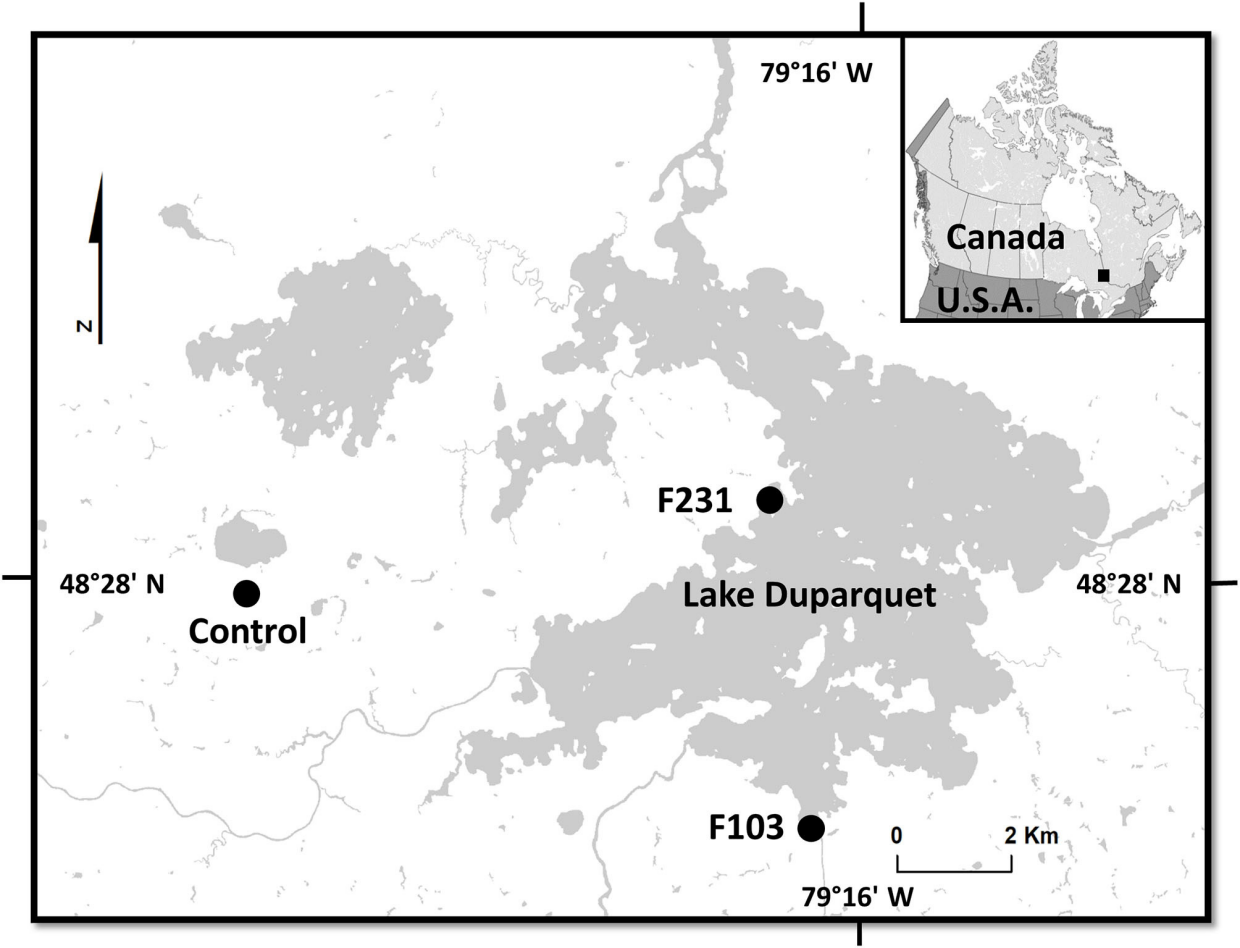
Map of Lake Duparquet showing the location of the sampled floodplain sites (F103 and F231) and the upland non-flooded control site (Control).

The study area is located in the Lake Duparquet region of north-western Quebec (Figure 1). The region is part of the Northern Clay Belt of Ontario and Quebec where rocky hills surrounding Lake Duparquet contain glacial till and lacustrine clay deposits (Daubois et al., 2015). The study area is situated in the mixed-wood boreal forest in the balsam fir-white birch domain (Bergeron et al., 1983). The nearest active weather station to the study area is located at Mont Brun (station 7085106), 41 km southeast of Lake Duparquet (https://climate.weather.gc.ca/). Between 1981 and 2010, the mean annual temperature was 1°C. The total annual precipitation was 985.2 mm with snowfall accounting for 28.5%. At Lake Duparquet, spring ice-breakup and subsequent flooding generally begin between mid-April and mid-May (Tardif and Bergeron, 1997a; Mongrain, 2014; Nolin et al., 2021a).

In the Lake Duparquet region, two sampling areas characterized by contrasting exposure to spring flooding were sampled. First, *F. nigra* stands were sampled along the floodplain of Lake Duparquet (48°28'N, 79°17'W; at an approximate elevation of 260 m above sea level; Figure 1). These stands were described in numerous studies (Tardif and Bergeron, 1992, 1999; Kames et al., 2016; Nolin et al., 2021a; Tardif et al., 2021). During extreme spring flood years like 1989 and 2019, *F. nigra* trees in the lower floodplain may have the lower portion of their stem remaining underwater into early July (Tardif, personal observation; Tardif et al., 2021). Second, a non-flooded *F. nigra* stand was sampled south of Lake Monsabrais and about 6 km east of Lake Duparquet (48°27'N, 79°25'W, approximate elevation 300 m above sea level, Figure 1). In this upland mesic site, *F. nigra* trees were not exposed to flood events and served as a control (hereafter referred to as control). Upland *F. nigra* stands in the study area have also previously been described (Bergeron et al., 1983; Tardif and Bergeron, 1997b; Kames et al., 2011). It should be noted that *F. nigra* prefers shallow or deep organic soils but also grows well on sandy soils underlain by clay that impedes drainage of water. The species is intolerant to severe drought due to its shallow root system and is adapted to mesic and hydric sites along streams, rivers, and lakes (Sims et al., 1990; Wright and Rauscher, 1990).

### Sample Collection and Preparation

In this study, we used *F. nigra* trees sampled by Kames (2009) and subsequently used in Kames et al. (2011, 2016). Detailed sampling and chronology development procedures can be found in the aforementioned publications. In each sampling area, wood samples were extracted as close to the tree base as possible using 5-mm diameter increment borers. Two cores were collected from each tree. At the laboratory, all cores were carefully mounted and glued on wooden supports. After drying, all cores were carefully sanded. After eliminating samples that were partly rotten and not suitable for image analysis, a total of 20 floodplain trees from two floodplain sites (Figure 1) and 21 trees from the control site (Figure 1) were retained for image analysis.

### Crossdating, Ring-Width Measurements, and Image Analysis

The date of tree-ring formation was determined by visual crossdating and matching tree-ring patterns based on the listing of unusually narrow and large rings (Phipps, 1985). Pointer years identified and chronologies developed for *F. nigra* in the Lake Duparquet region (Tardif and Bergeron, 1993, 1997b) were also used. After cross-dating, annual tree-ring widths in each core were measured with a VELMEX UniSlide measuring system (Velmex, Inc., Bloomfield, New York) and to a precision of 0.001 mm. The program COFECHA was used to validate cross-dating and measurement (Holmes, 1983).

Following initial ring-width measurement, samples were prepared for image analysis as described in the study of Tardif and Conciatori (2006). All cores were cleaned with pressurized air and rubbed with white chalk to increase the contrast between vessel elements and other cells. The surfaces were scanned with a Polaroid, USA, DMC digital camera connected to a Nikon Japan, SMZ stereo microscope to generate color images at a 25× magnification and a resolution of 1,600 × 1,200 pixels. Color images were analyzed with the program WinCell Pro (v. 2004a, Régent Instruments Inc., Quebec City, Canada, 2005) and the minimum cross-sectional lumen vessel area was set to 800 μm^2^ to capture all earlywood vessels. On each tree-ring image, the ring area was delimited, and the earlywood/latewood boundary was determined from the bimodal vessel size distribution in which larger-sized earlywood vessels can be distinguished from smaller-sized latewood ones (Panshin and de Zeeuw, 1980). The earlywood and latewood widths were also measured on each tree-ring image along with two radial files (lower and upper image portion) and averaged to obtain earlywood and latewood mean width which when summed provided mean annual ring width. These new ring-width measurements were validated with those obtained from direct measurements using the VELMEX (Velmex, Inc., Bloomfield, New York) system to control for potential mistakes. In this study, tree rings of very young cambial age, i.e., up to 10 years from the pith, were omitted from the analysis given their rather diffuse-porous vessel pattern.

### Chronology Developments

After data quality control, ring-width and vessel measurements were averaged by the tree (two cores) prior to deriving tree-ring anatomical traits. For each of the control and floodplain trees, nine variables were generated, namely, (1) earlywood width (EW), (2) latewood width (LW), (3) total ring width (RW), (4) earlywood total vessel cross-sectional lumen area (T), (5) earlywood number of vessels (N), (6) earlywood mean vessel cross-sectional lumen area (M), (7) earlywood mean cross-sectional area of the 25% largest vessels (25), (8) earlywood vessel density (d), and (9) earlywood porosity (p). Given that both the earlywood vessel fraction and lumen fraction are highly correlated to wood porosity (Ding et al., 2008), earlywood porosity was defined as the earlywood total vessel cross-sectional lumen area divided by the earlywood area. Density was defined as the number of vessels divided by the earlywood area. For each tree ring, the earlywood area was calculated by multiplying the ring area by the earlywood width and dividing the obtained value by ring width. Note that for chronologies generated for the control site, the capital letter C will be used whereas the capital letter F will be used for those originating from floodplain trees. In this study, the two flooded sites (Figure 1) were pooled to provide a single flood chronology given the excellent cross-dating (narrow, large, and flood ring) between sites (see also chronology statistics in Table 1). The study of Nolin et al. (2021b) also recently showed that the spring flood signal contained in earlywood vessels from floodplain *F. nigra* trees was coherent across four hydrological basins covering about 20,000 km^2^ in northeastern Canada.

**Table 1:** General statistics characterizing the standard (S) or residual (R) tree-ring widths and earlywood vessel chronologies generated from 21 and 20 trees from the control (C) and floodplain sites (F) respectively.

**Chronology**	**Mean** ^a^		**Mean sensitivity**		**Standard deviation**		**Auto-correlation** ^b^		**Variance in PC-1 (%)** ^c^		**EPS** ^c^		**Intertree correlation**		**Type** ^d^	
	**C**	**F**	**C**	**F**	**C**	**F**	**C**	**F**	**C**	**F**	**C**	**F**	**C**	**F**	**C**	**F**
EW (mm)	0.39	0.52	0.13	0.08	0.13	0.07	0.44	0.48	28	28	0.85	0.84	0.21	0.21	R	R
LW (mm)	0.65	0.56	0.44	0.39	0.37	0.36	0.27	0.12	59	45	0.96	0.92	0.56	0.36	R	S
RW (mm)	1.0	1.1	0.27	0.21	0.25	0.19	0.30	0.17	60	48	0.96	0.94	0.56	0.44	R	R
*N*	31	38	0.11	0.13	0.11	0.14	0.34	0.12	40	57	0.92	0.96	0.35	0.53	R	S
T (10^4^ μm^2^)	55	62	0.12	0.14	0.12	0.12	0.42	0.19	38	53	0.91	0.95	0.32	0.49	R	R
M (10^4^ μm^2^)	1.8	1.7	0.08	0.20	0.08	0.17	0.41	−0.05	29	67	0.87	0.97	0.24	0.64	R	S
25 (10^4^ μm^2^)	2.9	2.6	0.08	0.18	0.07	0.15	0.35	−0.05	26	67	0.84	0.97	0.20	0.63	R	S
d (10^−13^ mm^−2^)	1.8	1.6	0.09	0.10	0.08	0.12	0.16	−0.01	18	42	0.73	0.92	0.11	0.35	R	S
*p*	0.31	0.26	0.07	0.12	0.07	0.10	0.19	−0.06	15	52	0.56	0.95	0.06	0.47	R	S

*The chronologies for control and floodplain site trees cover the periods 1857–2006 and 1890–2006, respectively. All statistics, except when indicated, were calculated for these respective periods*.

*PC-1, first principal component; EPS, expressed population signal; EW, earlywood width; LW, latewood width; RW, ring-width; N, number of earlywood vessels; T, total cross-sectional vessel area of earlywood; M, mean cross-sectional vessel area of earlywood; 25, mean cross-sectional vessel area of the 25% largest earlywood vessels; d, vessel density in the earlywood and p, porosity in the earlywood*.

a *Calculated from measurement series*.

b *Calculated from standard chronologies*.

c *Calculated from the common period 1957–1998*.

d *Type of chronology used: Standard (S) or Residual (R). Note that standard chronologies were used when autocorrelation was not significant*.

To produce flood and control chronologies, time series pertaining to each of the 18 tree-ring variables were standardized using a cubic spline function with a 50% frequency response of 60 years. This procedure largely removed low-frequency variations associated with age-related trends or stands dynamics (Cook and Peters, 1981) while retaining high-frequency variability. Autoregressive modeling was only performed when autocorrelation was significant so all analyses were conducted using either standard or residual chronologies, i.e., with no significant serial autocorrelation. Each chronology was also developed using a biweight robust mean (Cook et al., 1990). To evaluate the statistical quality of each chronology, the mean sensitivity expressed population signal (EPS), percent variance of the first principal component, and between tree correlations were calculated. For all the above procedures, the program ARSTAN Windows (v. 4.0a; Cook, 1985) was used.

### Correlation Structure and Associations With Hydroclimatic Data

Prior to establishing correlations with hydroclimate data, principal components analysis (PCA) was performed on the 18 chronologies to explore their correlation structure and as a variable reduction procedure. All chronologies were equally weighted by using a correlation input matrix (Legendre and Legendre, 1998). All PCA was carried out using the period 1930–2005 which corresponded to a minimum of 10 trees being included in the chronologies.

In this study, the association between chronologies and hydroclimate variables was analyzed using two approaches. First, a PCA of the covariance matrix of solely significant correlation coefficients calculated between chronologies and hydroclimate variables was computed. For these correlation analyses, gridded monthly mean temperatures and total precipitation were obtained from the Climate Research Unit (CRU TS4.04; Harris et al., 2020; http://climexp.knmi.nl) for the 0.5° × 0.5° grid corresponding to Lake Duparquet (48.00°N, −79.50° E; 48.50N°, −79.00°E). The 3-month standardized precipitation evapotranspiration index (SPEI) derived from the CRU dataset was also used to assess drought impacts (Vicente-Serrano et al., 2010). The monthly record of snow cover extent from the Rutgers University Global Snow Lab (Estilow et al., 2015; https://climate.rutgers.edu/snowcover/) for the 1° × 1° grid corresponding to Lake Duparquet was also extracted. The monthly mean of the naturally flowing Harricana River daily discharge (station 04NA001-2; 48.57°N, −78.12° E) was also downloaded from the Reference Hydrometric Basin Network of Water Survey Canada (Water Survey of Canada, 2021; https://wateroffice.ec.gc.ca). We also made use of the global gridded runoff dataset (GRUN) providing monthly runoff time series on a 5° grid (Ghiggi et al., 2019). Second, the Climate Explorer engine (http://www.knmi.nl; Trouet and Oldenborgh, 2013) of the Royal Netherlands Meteorological Institute (KNMI) was used to calculate spatial field correlations between the first principal components obtained for each flood exposure (floodplain and control) and the individual surrounding grids. Unless specified, Spearman Rank Order correlations were calculated in all analyses and for the period 1930-2005, and all PCA were calculated using the program CANOCO v.5.12 (ter Braak and Šmilauer, 2018).

## Results

### Chronology Descriptive Statistics

Trees growing on control and floodplain had overall similar mean measurement values (Table 1). In floodplain trees, earlywood width however tended to be larger with earlywood vessels being slightly more numerous and smaller than in control trees. Width variables (EW, LW, and RW) had higher mean sensitivity and standard deviation values in control chronologies compared with the floodplain (Table 1). The inverse was however observed for each vessel chronology indicating higher year-to-year variations in floodplain trees. Comparing standard chronologies, each floodplain chronology except EW showed lower autocorrelation than control ones (Table 1). Statistics over the common period revealed that the floodplain vessel chronologies tended to share more common variance than the control ones and the inverse was true for width chronologies in control trees. For example, LW and RW in control trees recorded the highest values for variance accounted for by the first principal component (PC-1), the expressed population signal (EPS), and the mean inter-tree correlation. In contrast to the control, vessel chronologies from the floodplain shared the most variance. The floodplain vessel chronologies FM and F25 showed higher values for percent variance in PC-1, EPS, and Intertree correlation than did LW and RW of the control trees (Table 1). Overall all chronologies derived for control trees had a high common signal except earlywood vessel density and earlywood porosity (Table 1).

### Ring-Width, Vessel Chronologies, and Correlation Structure

**Figure 2:**
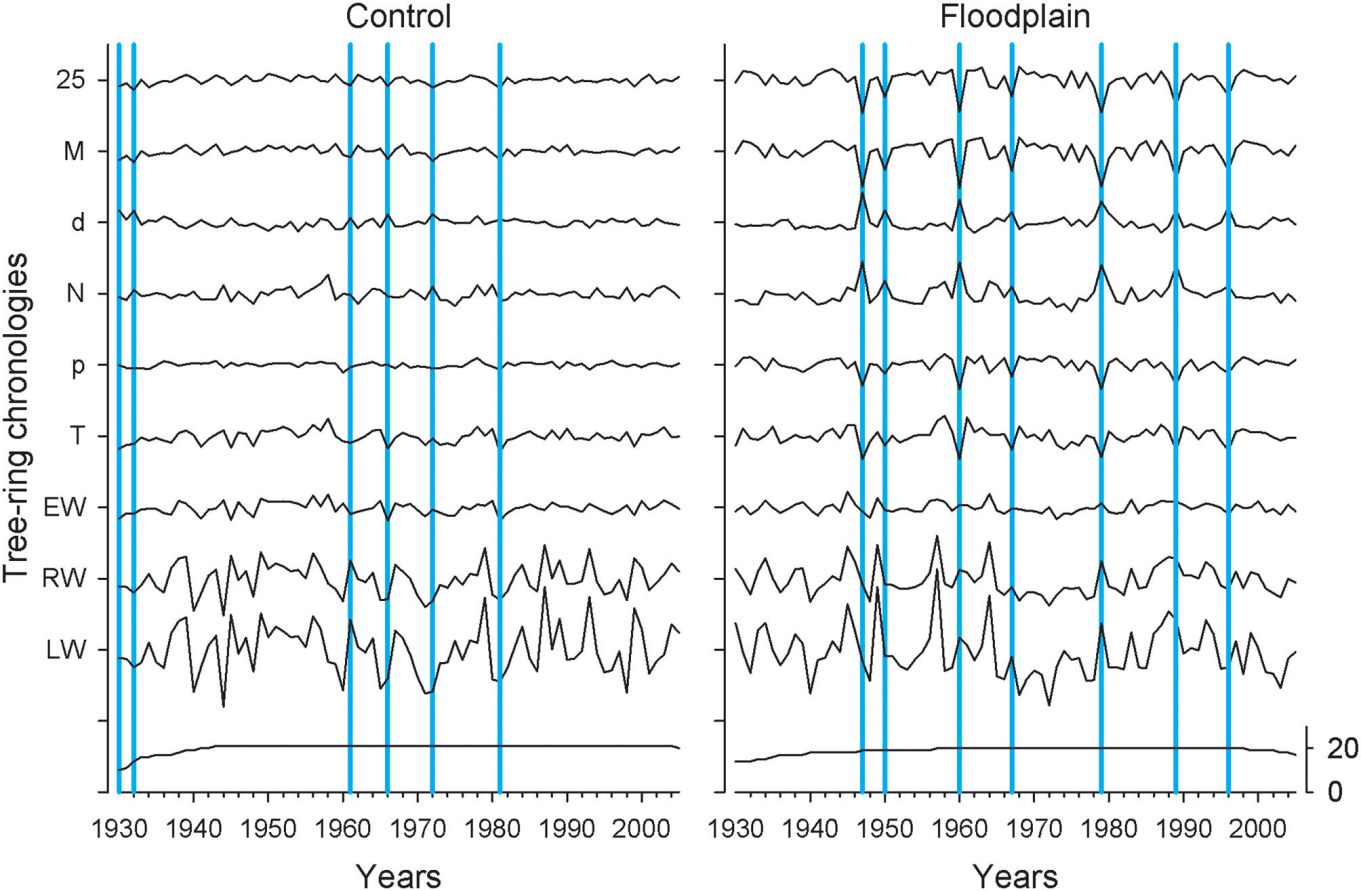
The 9 tree-ring chronologies for *Fraxinus nigra* growing on the control (left panel) and floodplain sites (right panel) with the number of samples indicated at the bottom (black line). The year 1930 corresponds to *F. nigra* chronologies in each flood exposure having a minimum of 10 trees. Years in which the earlywood mean vessel area (M) chronology deviated by −1.28 standard deviations are highlighted in blue. Chronologies are abbreviated as in Table 1.

Control and floodplain chronologies were generally dissimilar (Figure 2). The highest correlation observed between any two chronologies was for their respective latewood width (*rho* = 0.539, *p* < 0.001, *n* = 76). In both control and floodplain chronologies, LW and RW displayed the highest year-to-year variability (Table 1; Figure 2). In contrast to the control, all floodplain earlywood vessel chronologies presented much higher year-to-year variability with highly pronounced peaks and/or troughs observed over the period 1930–2005 (Figure 2). Years in which the floodplain mean vessel area chronology departed negatively from the mean by 1.28 standard deviations were 1947, 1950, 1960, 1967, 1979, 1989, and 1996. None of these years stood out in the corresponding control chronology. In the control, years that deviated negatively by at least 1.28 standard deviations from the mean vessel area chronology were 1930, 1932, 1961, 1966, 1972, and 1981 (Figure 2). Common years in which the respective control and floodplain mean vessel area chronology departed positively from the mean by 1.28 standard deviations were 1943, 1959, 1962, and 1968.

**Figure 3:**
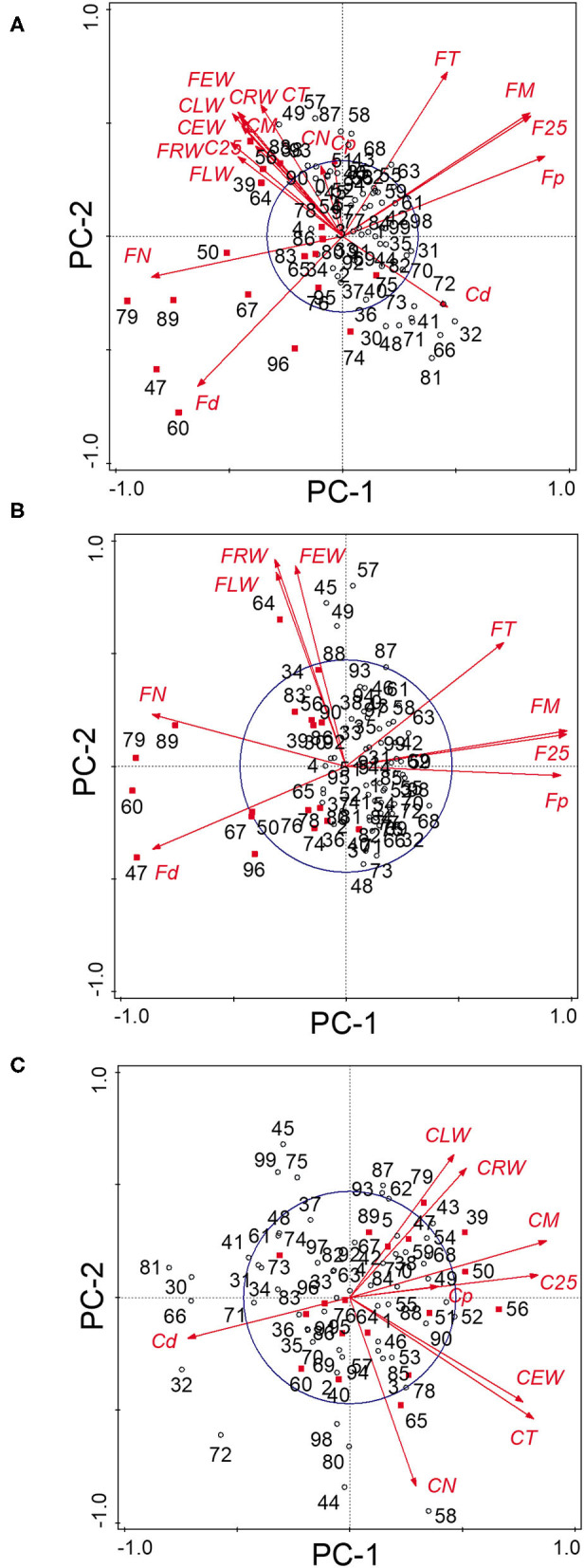
Principal components analysis of the **(A)** 18 tree-ring chronologies for the control and floodplain sites, **(B)** for the floodplain site, and **(C)** for the control site. The first two principal components axes are shown. The capital letter preceding each chronology abbreviation indicates their origin: control (C) or floodplain (F). Chronologies are abbreviated as in Table 1. The reference period is 1930–2005 with years marked in red indicating major flood-ring years (Tardif et al., 2021). The circle of equilibrium is indicated on each sub-figure.

**Figure 4:**
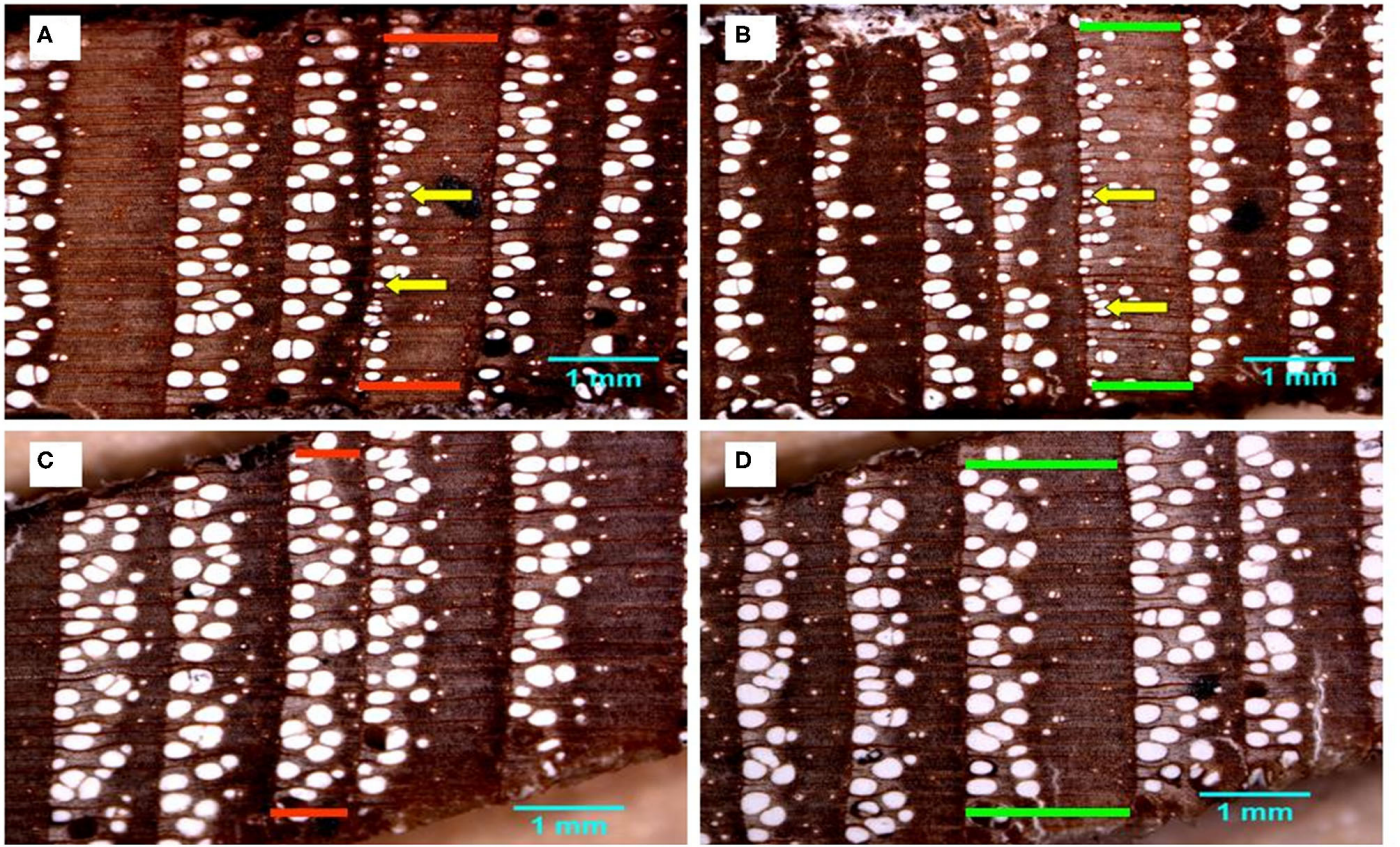
*Fraxinus nigra* tree rings from floodplain **(A,B)** and control **(C,D)** trees showing rings of the years 1960 and 1979 delineated in red and green, respectively. Yellow arrows point to examples of earlywood vessels with largely reduced lumen area and characteristic of macroscopically defined flood rings.

The PCA of the 18 chronologies (Figure 3) confirmed the weak common variance between floodplain and control chronologies and especially of their vessel chronologies (Figure 3A). The first two components totalized 53.8% of the variance with PC-1 and PC-2 representing respectively 30.6 and 23.2% of the total variance. The first PC was strongly associated with floodplain earlywood vessel chronologies (Figure 3A). Floodplain vessel number (FN) displayed a strong negative correlation with PC-1 with vessel dimensions (FM, F25, and Fp) being positively correlated with it. The acute angle between vectors pertaining to floodplain earlywood vessel chronologies indicates strong positive correlations and both FN and Fd chronologies were negatively associated with FM, F25, FT, and Fp. Major flood years, e.g., 1960 and 1979, also had negative loadings on PC-1. In these years, floodplain trees recorded a high number of earlywood vessels (density) of small size (Figures 3A, 4A,B). In contrast, the control trees recorded larger vessels in both 1960 and 1979, with 1960 being a year with thin latewood (Figures 4C,D). The second PC was positively associated with both control and floodplain width chronologies and, with control vessel dimension (Figure 3A). Floodplain earlywood total vessel cross-sectional lumen area had the highest loading on PC-2. It should be noted that a robust-PCA calculated from a Spearman's correlation matrix, and another calculated after eliminating the years for which the floodplain mean vessel area chronology departed negatively from the mean by at least 1.28 standard deviations, revealed essentially the same structure (not presented) indicating that the differences observed between flood exposures were not driven by a few extreme values.

Given the distinct signal originating from floodplain and control chronologies, a PCA was recalculated for each hydrological context (floodplain and control) to get a finer view of the intricate linkages among earlywood attributes. For the floodplain, the first two PCs explained 88.5% of the total variance with PC-1 and PC-2 capturing, respectively, 56.1 and 32.4% of the variance (Figure 3B). Again, PC-1 evidently reflected its strong association with earlywood vessel chronologies and PC-2 with ring-width chronologies (EW, LW, and RW). The total vessel area chronology from the floodplain also loaded high on PC-2 as did the large tree rings of 1945, 1949, 1957, and 1964. While vessel size attributes were strongly correlated with one another as shown by their acute and/or obtuse angles, they were decoupled from width attributes as shown by the nearly right (90°) angle between respective vectors (Figure 3B). Flood years were generally characterized by numerous and small earlywood vessels in floodplain trees (Figures 4A,B). For the control PCA, the first two PCs explained, respectively, 44.2 and 22.8% of the total variance (Figure 3C). The third PC (not presented) expressed 17.1% of the variance and was mainly related to latewood and total ring width. Similar to the floodplain site, PC-1 of the control site was negatively associated with vessel density (Cd) and positively with earlywood vessel dimensions (CM, C25, and CT; Figure 3C). However, in the control site, width attributes had a positive loading on PC-1 as well as the number of earlywood vessels. Earlywood width and total vessel area were also strongly correlated as indicated by the acute angle between their respective vectors (Figure 3C). Years with thicker earlywood were characterized by a greater total vessel area (CT); a trait not observed on the floodplain site (Figure 3B). On the control site, earlywood width was most strongly correlated with earlywood total vessel area (*rho* = 0.826, *p* < 0.001, *n* = 76); this correlation dropped in the flooded site (*rho* = 0.252, *p* = 0.028, *n* = 76). Interestingly, some flood-ring years, e.g., 1950; 1956, also corresponded to years with thick earlywood and high mean vessel area. The spread of the identified flood-ring years in the ordination plane however strongly differs from that of the floodplain (Figures 3B,C). The flood year 1960 and 1979, respectively, led to narrow and thick rings in the control site (Figures 3C, 4C,D). The respective PC-1 calculated from the nine control and the nine floodplain chronologies (Figures 3B,C) were not significantly associated (*rho* = −0.102, *p* = 0.381, *n* = 76) despite each representing variance contained in their respective earlywood vessel chronologies. A significant but small correlation was however observed between control PC-1 and floodplain PC-2 (*rho* = 0.255, *p* = 0.026, *n* = 76), wherein both axes accounting for variance, among others, related to ring-width attributes (EW, LW, and RW).

### Associations Between Chronologies and Climate Data

**Figure 5:**
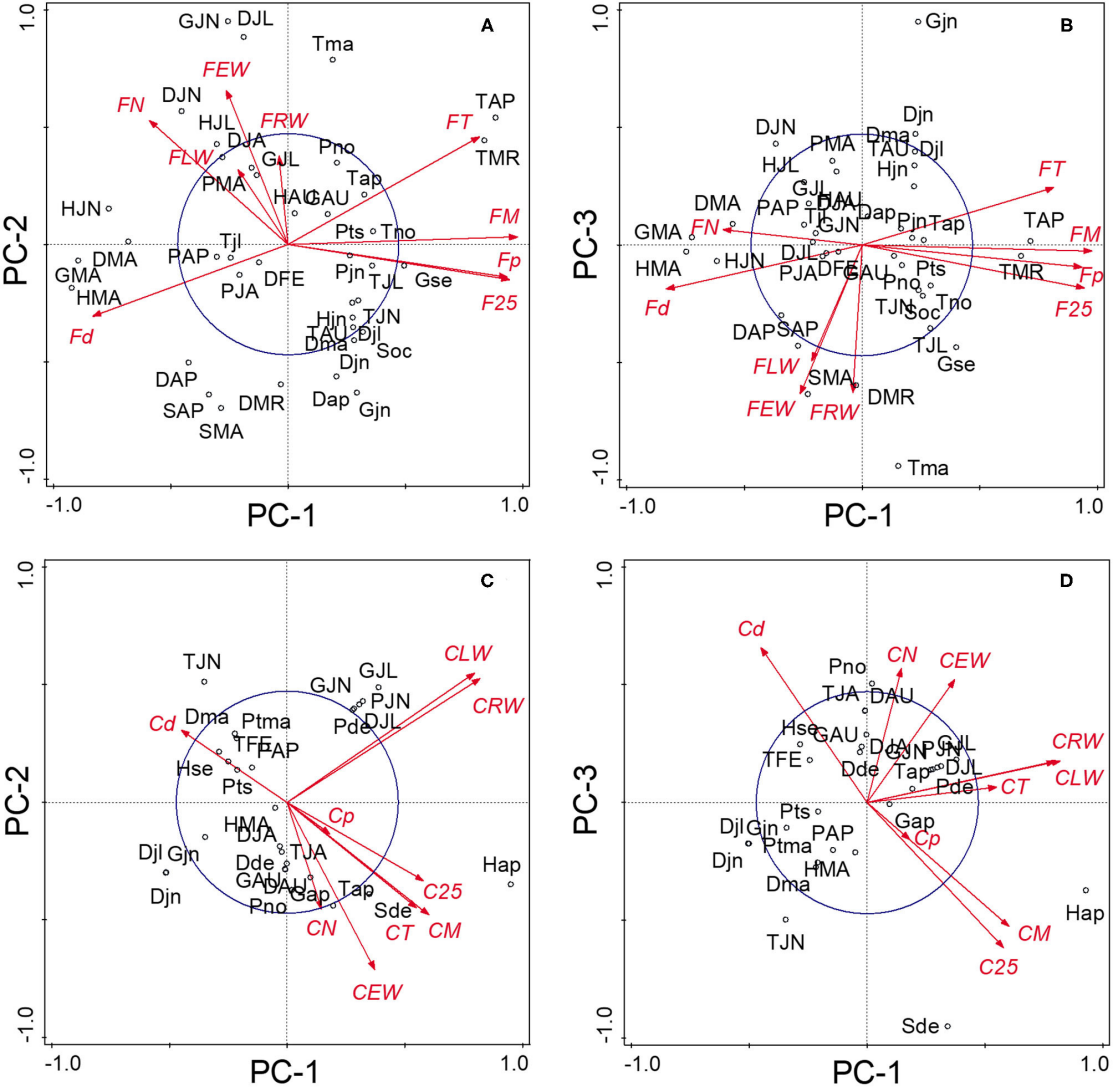
Principal components analysis calculated from significant (*p* < 0.05) Spearman's rho correlation coefficients between chronologies and hydroclimatic variables. The first two principal components for the floodplain site are shown in **(A)** and components 1 and 3 are shown in **(B)**. The first two principal components for the control site are shown in **(C)** and **(D)** represents the first and third components. T, temperature; P, precipitation; S, snow cover; D, SPEI-3; H, Harricana River discharge; G, GRUN runoff. Months are identified by their first two letters with capital letters indicating the current growing season. Chronologies are abbreviated as in Table 1. The circle of equilibrium is indicated on each sub-figure.

In continuity with the previous analyses, the floodplain and control chronologies showed distinct patterns in their correlations with selected hydroclimate variables. First, the control chronologies were significantly (*p* < 0.05) associated with 26 variables out of 93 candidate ones whereas the floodplain ones were significantly associated with 40 candidate variables. Second, the control chronologies had 51 significant (*p* < 0.05) correlation coefficients compared with 124 for the floodplain chronologies. Third, the significant correlation coefficients from the control chronologies had a smaller range [from −0.414 to 0.392] compared with floodplain ones [from −0.696 to 0.458]. The PCA calculated from the significant correlation coefficients indicated a distinctive response between flood exposures (Figure 5).

For the floodplain site, the first three PCs explained, respectively, 73.0, 9.9, and 5.5% of the total variance (Figures 5A,B). The ordination plan (Figure 5A) was very similar to that obtained from the chronologies (Figure 3B). The acute angle between earlywood vessel chronologies, e.g., FM, Fp, and F25, indicated that they shared similar correlations with hydroclimate variables. The near 90° angles between vessels and width variables (exception of the number of vessels) also illustrated the aforementioned decoupling between ring-width and earlywood vessel dimensions in floodplain trees (Figure 5A). The first PC was associated with vessel dimension, e.g., total and mean vessel area, and these attributes were associated with the May and June Harricana discharge (negative association) and March and April temperature (positive association). These associations were inverted with vessel density. The highest correlation coefficient (−0.696, *p* < 0.01, *n* = 76) was obtained between the mean vessel area and the May Harricana discharge. The may GRUN runoff was also negatively associated with vessel dimensions. The high April-May-June SPEI-3 (especially May), indicating wetness, was also negatively associated with earlywood vessel dimensions (Figure 5A). The negative influence of hydric conditions was further emphasized by the negative association between the April and May snow cover and earlywood vessel dimensions, e.g., mean vessel area: −0.426 and −0.384, respectively; vessel density: 0.458 and 0.403, respectively, *p* < 0.05, *n* = 36. For the floodplain site, PC-2 was mainly associated with earlywood width and vessel number, and to a lesser extent, with latewood and total ring width (Figure 5A). These variables were positively associated with the June GRUN runoff and both June-July SPEI-3. The may temperatures of the previous year were also positively associated with the variables. The June GRUN runoff of the previous year as well as the March-April SPEI-3 of the current year was also negatively associated with PC-2. Little variance (5.5%) was associated with PC-3 with earlywood and total ring width being mainly associated positively with the May temperature of the previous year and negatively with the June GRUN runoff of the previous year (Figure 5B).

Similar to floodplain chronologies (Figure 3B) approximating that of the correlation coefficient with hydroclimate variables (Figure 5A), both PCAs of the control site (Figures 3C, 5C) were similar with all vectors (except vessel density) having a positive loading on PC-1. The first three PC explained respectively 35.8, 27.2, and 19.2% of the total variance indicating that tree-ring attributes were less severely modulated by a single set of hydroclimate variables. No significant negative correlations with the May-June GRUN runoff or Harricana discharge of the current year were observed. In contrast, the June and July GRUN runoff were positively associated with PC-1. The first PC largely reflected the common variance in latewood (total ring) width, and to a lesser extent, with earlywood vessel attributes. PC-2 separated earlywood characteristics (width and vessels) from latewood (total ring) width. The April Harricana discharge of the previous year was positively associated with PC-1 and to the production of thick tree rings and large earlywood vessels (Figure 5C). More specifically, large rings were positively associated with precipitation in December and June, with the June-July GRUN runoff, and July SPEI-3. The June-July SPEI-3 of the previous year was negatively associated with latewood (total ring) width. Earlywood width and vessel dimensions were negatively associated with the June temperature and positively with the December snow cover extent (Figure 5B). The third PC mainly separated earlywood width (and the number of vessels) from mean vessel area (and largest vessels), wherein the latter being positively associated with December snow cover extent [*rho* = 0.381 (and.392), *p* < 0.05, *n* = 36]. The number of earlywood vessels was negatively associated with the current June temperature (*rho* = −0.298, *p* < 0.05, *n* = 76).

**Figure 6:**
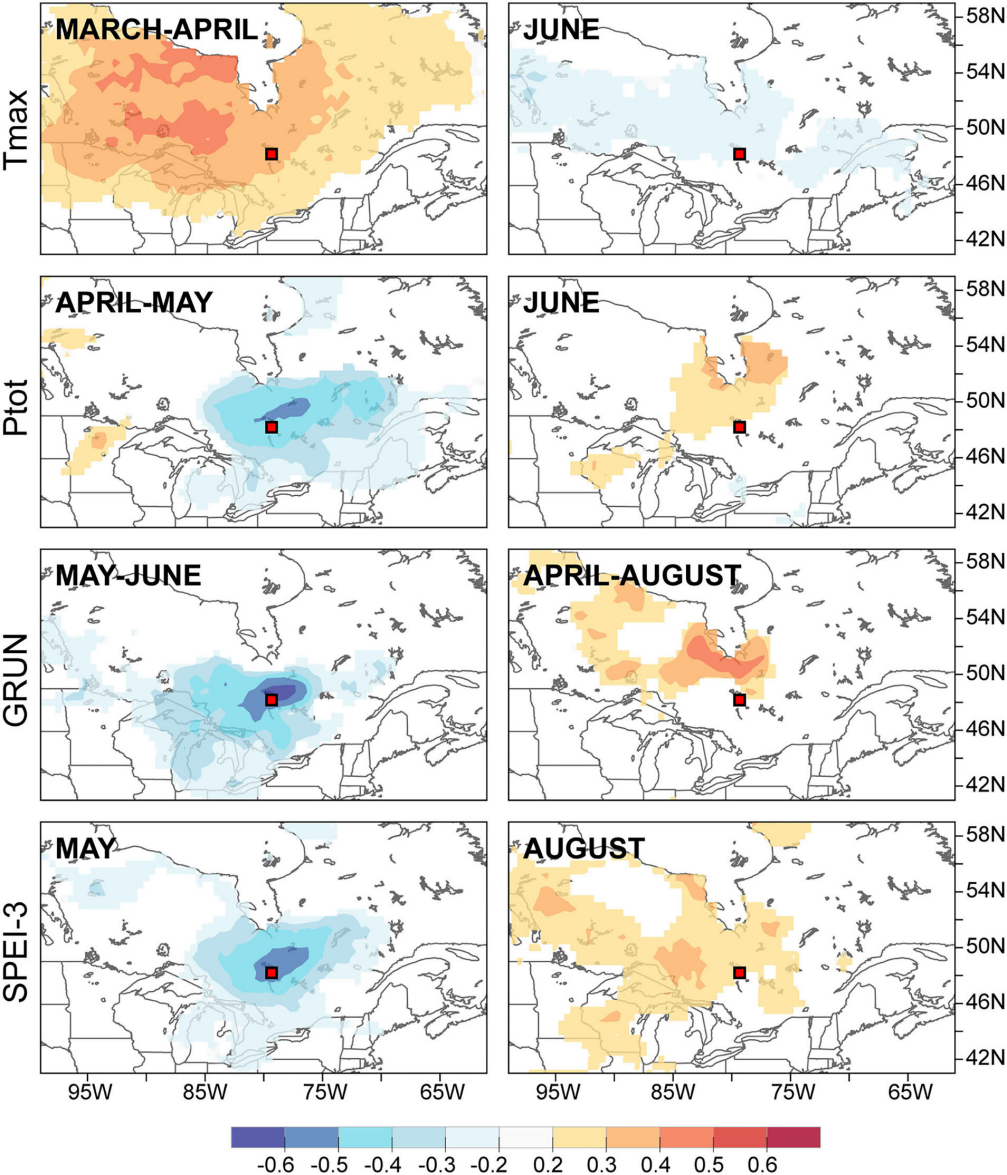
Spatial field correlation between the first principal components extracted from the floodplain chronologies (left column) and the control chronologies (right column) and selected hydroclimate variables.

The spatial field correlations calculated between the PC-1 yearly loadings of the chronologies (Figures 3B,C) and selected hydroclimatic variables further highlighted the distinct but spatially coherent signal in trees from both flood exposures (Figure 6). Floodplain trees with tree rings with large earlywood vessel dimensions (FM, F25, and Fp) were positively associated with the warm March-April temperatures, low April-May precipitation, low May-June runoff, and low May SPEI-3 values. In other words, flood rings were favored by cold and wet springs with high runoff and high (wet) SPEI-3 values (Figure 6). This signal was spatially coherent over a large portion of northeastern Ontario and northwestern Quebec. In contrast, few and weak spatially coherent associations were found with PC-1 of the control site. Among them, the June maximum temperature was negatively associated with PC-1 (inverse with June precipitation). In contrast to the floodplain, the control site displayed a positive correlation with the April-August average GRUN runoff north of the study area (Figure 6). The August SPEI-3 was also positively associated with PC-1. In other words, thick tree rings with large vessels were mainly associated with abundant runoff and a lack of drought during the growing season in the control site.

## Discussion

The differences between flooded and non-flooded control *F. nigra* trees could be systematically observed from their chronology statistics, cross-correlation structure, and distinct associations with hydroclimate variables. Some tree-ring traits transcended flood exposure like the lower year-to-year variability in EW compared with LW and RW which has been reported in numerous ring-porous species growing in various habitats (Tardif, 1996; Corcuera et al., 2004; Fonti and García-González, 2004, 2008; Tardif and Conciatori, 2006; Davis and Loader, 2020). The low EW year-to-year variability in ring-porous species may reflect strong developmental controls. Similarly, the lower common signal strength and mean sensitivity of earlywood vessel vs. width chronologies (observed more strongly in control trees) were also reported in numerous ring-porous species (Woodcock, 1989; Fonti and García-González, 2004; Tardif and Conciatori, 2006; Alla and Camarero, 2012; García-González et al., 2016; Pritzkow et al., 2016). However, compared with the control, the floodplain earlywood vessel chronologies generally showed greater common signal strength including higher mean sensitivity and a stronger response to hydroclimate factors.

Another noticeable difference between control and floodplain trees was related to the thicker EW observed in the latter and its greater contribution to RW. Floodplain trees may experience a lengthened leaf development period compared with control trees making earlywood production last longer than in the control site. Studies indicated that in ring-porous species earlywood may cease to be produced 1–2 weeks after shoot extension and complete leaf expansion (Chalk, 1930; Zasada and Zahner, 1969). Recent studies have also shown that the relation between vessel formation and leaf phenology may vary within and between ring-porous species (Sass-Klaassen et al., 2011; Puchałka et al., 2017). In the 5-year study Ahlgren (1957) reported that *F. nigra* trees growing in northeastern Minnesota started radial growth between the first to the third week of May and that the first leaves in recognizable form were observed between the 3rd week of May and the 1st week of June. In southeastern Ontario, the wood formation was also initiated in *F. nigra* at the beginning of May (Fraser, 1958). In major flood years, leaf development in floodplain *F. nigra* trees is delayed and/or slower than in non-flood years (Tardif pers. observation). Xylogenesis and phenological monitoring of flooded and non-flooded trees along a timeframe including years with low and high flood magnitude may help to better clarify these habitat-specific differences (Sass-Klaassen, 2009; Sass-Klaassen et al., 2010).

### Differences Among Control and Floodplain Chronologies

Another striking difference between control and flooded chronologies was the decoupling of vessel attributes from earlywood width in the latter. In the control site, the strong positive associations of EW with vessel number and total vessel area have been reported in numerous ring-porous species growing in various non-riparian habitats (Fonti and García-González, 2004; Tardif and Conciatori, 2006; González-González et al., 2015; Zhu et al., 2020). Environmental conditions that favored large EW at the control site also promote the production of numerous somewhat larger-sized vessels resulting in a high total vessel area with low earlywood vessel density. In contrast, environmental conditions inducing large EW on the floodplain also brought an increase in vessel number but not in vessel dimension (M, T). On the floodplain, earlywood characterized by a high number of vessels tended to contain small-sized vessels culminating in a low total vessel area. These rather “diffuse-porous” earlywood vessel patterns were unique to floodplain trees, most pronounced in years of high magnitude spring flood and not observed in control trees. These results further confirm that in the floodplain trees, the production of a higher number of vessels of smaller areas is associated with spring flood duration. As hypothesized, vessel chronologies from non-flooded trees were independent from those developed from flooded trees confirming the uniqueness of the flood signal to floodplain trees.

In contrast to earlywood vessels, ring-width attributes showed to be positively correlated between flood exposures indicating some shared variance. Tardif and Bergeron (1997b) which used different sites also reported as in this study a lack of clear cross-dating between the floodplain and upland *F. nigra* trees with their total ring width chronologies showing a low correlation (*r* = 0.32, *p* < 0.01). It is speculated that more similar growth conditions may occur in years of low flood magnitude (relaxing of flood influence) with tree-ring development (EW and LW) being more similarly affected by the prevalent climate conditions than during major flood years. Again, xylogenesis and phenological monitoring of flooded and non-flooded trees over a range of flood years may help clarify why ring-width attributes show more between habitats similarities than vessel ones. In a recent study comparing upper- and lower-floodplain trees, Tardif et al. (2021) observed that flood rings almost disappeared in upper-floodplain trees compared with lower-floodplain trees. While the authors did not report on ring width, total ring width chronologies developed for the upper and lower floodplain sites showed to be significantly and positively correlated (result not presented). In a similar way, the study of Nolin et al. (this issue) reported that flood rings became less frequent and harder to distinguish in riparian *F. nigra* trees after hydrological regulation with little impact noticeable in total ring width among sites. These results suggest that earlywood vessels attributes (number and mean area), in contrast to tree-ring width, could be used to assess the impact of hydrological regulation and/or to maintain (restore) spring flood conditions within their historical range of variability in managed systems.

In this study, flooding occurring during the active growing season was presumably the most important factor influencing the number, density, and especially the mean and total area of the earlywood vessels in floodplain trees. Of all the vessel attributes measured, earlywood mean vessel area and vessel number were the most strongly associated with discharge (Kames et al., 2016; Nolin et al., 2021a). The lack of negative correlation between the control earlywood vessel chronologies and spring river discharge further reinforces the hypothesis that flooding exerts a strong influence on earlywood vessel formation. The large decrease in earlywood mean vessel area in high magnitude flood years observed in this study was consistent with observations made for *F. americana, F. pennsylvanica* (Yanosky, 1983), *Q. macrocarpa* (St. George and Nielsen, 2000, 2003), and *Q. robur* (Astrade and Bégin, 1997; Sass-Klaassen, 2009, Copini et al., 2016). Compared with these studies, a unique feature in the response of *F. nigra* to flooding was the increase in the number of earlywood vessels. It is possible that the specific response of *F. nigra* may be associated with its strong tolerance to flooding (Sims et al., 1990). Comparisons with other species are further complicated by the fact that the study of Yanosky (1983) did not develop continuous vessel chronologies, while the study of Astrade and Bégin (1997) developed only a short time series in which the number of vessels was not assessed.

As previously proposed, the decrease in mean vessel area and the increase in the number of vessels in response to spring flooding may be related to alterations in growth regulator dynamics including both ethylene and auxin which act on xylem development (Yamamoto and Kozlowski, 1987; Yamamoto et al., 1987; Aloni, 2015). An interplay of both, auxin and ethylene on vessel size is supported through studies with hybrid poplars (*Populus tremula* L. × *Populus tremuloides* Michx. and *Populus tremula* L. *x Populus alba* L.), white poplar (*Populus alba* L.), as in these species large increases in the concentrations of auxin and ethylene led to reductions in vessel area (Tuominen et al., 1995; Junghans et al., 2004). A decreased vessel area was also observed following the application of the ethylene releasing compound ethrel in American elm (*Ulmus americana* L.) (Yamamoto et al., 1987) and Norway maple (*Acer platanoides* L.) (Yamamoto and Kozlowski, 1987). In *U. americana*, ethrel application also caused an increase in the number of vessels (Yamamoto et al., 1987). Further, ethylene was reported to decrease the vessel diameter in Manitoba maple (*Acer negundo* L.; Savidge, 1988).

While not studied, preliminary observations indicate that vessel grouping [see von Arx et al. (2013)] may be altered during years with high magnitude spring flood. Tardif et al. (2021) noted that flooded *F. nigra* trees often produced vessel chains instead of solitary vessels during flood years. This response may however not be systematic (see Figure 2) and it raises the question whether alterations in vessel connectivity could be traced back to either core sampling height and/or to the duration of stem submersion. Answering this question may provide added value to flood rings as a hydrological proxy. Detailed analysis of specific flood years (accompanied by thin sectioning) may provide further insights on the impact of flooding on *F. nigra* growth. In this study, the impact of flooding on latewood characteristics was not investigated. The study of Yamamoto et al. (1995) observed a reduction in the cell wall thickness of wood fibers following a flood treatment of Manchurian ash (*Fraxinus mandshurica* Rupr. var. *japonica* Maxim.) seedlings. In floodplain habitat, neglecting small vessels (< 6,000 or < 10,000 μm^2^) as often recommended in numerous studies (García-González and Fonti, 2006; for examples, see García-González and Fonti, 2006; Fonti et al., 2009a; González-González et al., 2014; García-González et al., 2016) may prove prejudicial and thresholds smaller than 800 μm^2^ may be needed to better quantify the impact of flooding on latewood characteristics in species like *F. nigra*.

### Different Responses to Hydroclimatic Factors

The correlations between chronologies and hydroclimate variables displayed by the floodplain and control *F. nigra* trees were as contrasted as their chronologies. The results of this study supported the hypothesis that earlywood vessel chronologies from non-flooded trees show lesser potential for hydroclimate reconstruction compared with their floodplain counterparts. Vessel attributes in floodplain trees strongly responded to hydrological signal of Lake Duparquet. Years with increasing May and June mean Harricana River discharge (also May and June GRUN runoff) were strongly associated with tree rings with more numerous but reduced sized earlywood vessels. Vessel chronologies were also essentially correlated with climate factors leading to high magnitude spring floods, i.e., cool March and April temperatures leading to a persistent snow cover in the spring. These findings are in line with previous *F. nigra* studies (Tardif et al., 2010, 2021; Kames et al., 2016; Nolin et al., 2021a,b). The results from these studies all converged, revealing the major role played by flood magnitude (timing and duration) on tree-ring development in floodplain trees.

These results evidently contrast with those obtained from the control site in which vessel chronologies and spring discharge were not significantly correlated. Control *F. nigra* trees displayed a different signal than floodplain trees with surprisingly no strong drought signal observed. Significant correlations with hydroclimate variables were few and generally weak. In the control site, the mean earlywood vessel area solely showed a weak correlation with the December snow cover (positive) and mean February temperatures (negative), suggesting that a sufficient amount of water may be supplied by snowmelt during the earlywood vessel production period. The June and July GRUN runoff were positively associated with large tree rings as well as the June precipitation and July and August SPEI-3, indicating the importance of water availability during the growing season. Despite *F. nigra* trees exhibiting determinate growth (Lechowicz, 1984) with winter buds containing all leaf primordia that will develop in the next growing season (Pallardy, 2008), previous year conditions did not show to be strongly associated with the current year growth. In both flood exposures, conditions in the prior growing season including warm spring (April for control and May for floodplain) and the reduced June GRUN runoff could indicate that early breakage of dormancy and a long growing season may allow for greater carbohydrates production to be used in the following year earlywood production.

In both flood exposures, ring-width attributes were correlated to a different set of hydroclimatic variables compared with vessel attributes. In floodplain trees, width attributes contrary to vessels showed little correlation to spring discharge data and revealed to be lower quality hydrological proxies. Using *F. nigra* ring-width chronology only, the study of Tardif and Bergeron (1997b) had also stressed major differences between upland and floodplain regarding their climate associations. In this study, the absence of a significant association between the floodplain EW and May (t) discharge was unexpected. The study of Tardif (1996) has found a weak but significant negative relationship between the EW of floodplain *F. nigra* growing on the floodplain of the Lake Duparquet. *Fraxinus nigra* trees analyzed by Tardif (1996) were however growing at a very low elevation and more exposed to flooding than trees analyzed in the present study (Tardif and Bergeron, 1992, 1999). In floodplain habitats, small differences in elevation (also coring height) may affect tree-ring attributes and their association with hydrological (Tardif and Bergeron, 1993; Nolin et al., this issue; Tardif et al., 2021). In this context, extracting cores at 1.3 m and/or excluding small earlywood vessels in floodplain ring-porous species may not be desirable when maximizing flood signal is the objective.

## Conclusion

The objectives of this study were (i) to describe and compare ring-width and earlywood vessel chronologies in *F. nigra* trees growing in two contrasting exposure to spring floods and (ii) to determine their degree of association to hydroclimatic variables. The clear distinctions between flooded and non-flooded chronologies were maintained from their descriptive statistics to their associations with hydroclimate variables. In control trees, the linkage between earlywood width and vessel characteristics was much more pronounced than in floodplain trees. In control of *F. nigra* trees, conditions leading to large EW promote the production of numerous and large-sized vessels, thus resulting in a higher mean and total vessel area. In floodplain trees, this linkage between earlywood width and vessel size was weak and the most important factor influencing vessel number and especially mean and total vessel area was spring flood magnitude. Control trees, compared with floodplain ones, displayed no negative associations with spring river discharge. The weak and rare correlations displayed by control trees support the idea that vessel chronologies from trees growing in sites that are not highly “stressed” may not be worth developing for dendroclimatological purposes.

Given the unambiguous influence of spring floods on floodplain *F. nigra* trees, future work may focus more actively on them. In floodplain habitat, a threshold smaller than 800 μm^2^ (accompanied by thin sectioning) may be needed to further quantify the impact of spring floods on latewood characteristics. Detailed tree-ring analyses of specific flood years would allow measuring alterations in both vessels and fibers. Looking at changes in vessel connectivity may also be of interest. Detailed studies may also help elucidate why mean vessel area chronology over- and/or underestimate flood magnitude in some years. Given that flood rings appear to be essentially restricted below the flood-water line during vessel formation; stem analysis may prove useful to determine if *in-situ* spring water levels can be reconstructed. Finally, the observed earlywood vessel plasticity in floodplain *F. nigra* trees suggests that it could be used to (i) evaluate flood regime alterations following regulation and/or (ii) restore/maintain flooding within its historical range assuming reference trees are available. Further studies are also required that investigate vessel chronologies in other floodplain (ring- and diffuse-porous) species and under various hydrological contexts.

## Data Availability Statement

The raw data supporting the conclusions of this article will be made available by the authors, without undue reservation.

## Author Contributions

JT, SK, and YB contributed to conception and design of the study. SK and JT performed sampling. SK performed measurements and organized the database. JT, SK, and AN performed formal analyses and data interpretation. JT and YB supervised and provided funding for the project. JT and SK wrote the first draft of the manuscript. All authors provided critical feedback, contributed to manuscript revision, read, and approved the submitted version.

## Funding

This research was funded by a NSERC-Discovery grant to YB and a NSERC-Discovery grant to JT.

## Conflict of Interest

The authors declare that the research was conducted in the absence of any commercial or financial relationships that could be construed as a potential conflict of interest.

## Publisher's Note

All claims expressed in this article are solely those of the authors and do not necessarily represent those of their affiliated organizations, or those of the publisher, the editors and the reviewers. Any product that may be evaluated in this article, or claim that may be made by its manufacturer, is not guaranteed or endorsed by the publisher.
